# Autophagy-Associated IL-15 Production Is Involved in the Pathogenesis of Leprosy Type 1 Reaction

**DOI:** 10.3390/cells10092215

**Published:** 2021-08-27

**Authors:** Bruno Jorge de Andrade Silva, Tamiris Lameira Bittencourt, Thyago Leal-Calvo, Mayara Abud Mendes, Rhana Berto da Silva Prata, Mayara Garcia de Mattos Barbosa, Priscila Ribeiro Andrade, Suzana Côrte-Real, Gilberto Marcelo Sperandio da Silva, Milton Ozório Moraes, Euzenir Nunes Sarno, Roberta Olmo Pinheiro

**Affiliations:** 1 Leprosy Laboratory, Oswaldo Cruz Institute, Oswaldo Cruz Foundation, FIOCRUZ, Rio de Janeiro 21040-360, Brazil; brunojas85@outlook.com (B.J.d.A.S.); tamiris.lameira@gmail.com (T.L.B.); thyagoleal@yahoo.com (T.L.-C.); mayarah.rj@gmail.com (M.A.M.); rhana.prata@gmail.com (R.B.d.S.P.); may.barbosa.87@gmail.com (M.G.d.M.B.); prandrade86@gmail.com (P.R.A.); milton.moraes@fiocruz.br (M.O.M.); euzenir.sarno@fiocruz.br (E.N.S.); 2 Structural Biology Laboratory, Oswaldo Cruz Institute, Oswaldo Cruz Foundation, FIOCRUZ, Rio de Janeiro 21040-360, Brazil; scrf@ioc.fiocruz.br; 3 Evandro Chagas National Institute of Infectology, FIOCRUZ, Rio de Janeiro 21040-360, Brazil; gilbertomarcelo@gmail.com

**Keywords:** autophagy, *Mycobacterium leprae*, leprosy or T1R, macrophage or THP-1, phagosome, lysosomes, IL-15

## Abstract

Leprosy reactional episodes are acute inflammatory events that may occur during the clinical course of the disease. Type 1 reaction (T1R) is associated with an increase in neural damage, and the understanding of the molecular pathways related to T1R onset is pivotal for the development of strategies that may effectively control the reaction. Interferon-gamma (IFN-*γ*) is a key cytokine associated with T1R onset and is also associated with autophagy induction. Here, we evaluated the modulation of the autophagy pathway in *Mycobacterium leprae*-stimulated cells in the presence or absence of IFN-*γ*. We observed that IFN-*γ* treatment promoted autophagy activation and increased the expression of genes related to the formation of phagosomes, autophagy regulation and function, or lysosomal pathways in *M. leprae*-stimulated cells. IFN-*γ* increased interleukin (IL)-15 secretion in *M. leprae*-stimulated THP-1 cells in a process associated with autophagy activation. We also observed higher *IL15* gene expression in multibacillary (MB) patients who later developed T1R during clinical follow-up when compared to MB patients who did not develop the episode. By overlapping gene expression patterns, we observed 13 common elements shared between T1R skin lesion cells and THP-1 cells stimulated with both *M. leprae* and IFN-*γ*. Among these genes, the autophagy regulator Translocated Promoter Region, Nuclear Basket Protein (*TPR*) was significantly increased in T1R cells when compared with non-reactional MB cells. Overall, our results indicate that IFN-*γ* may induce a TPR-mediated autophagy transcriptional program in *M. leprae*-stimulated cells similar to that observed in skin cells during T1R by a pathway that involves IL-15 production, suggesting the involvement of this cytokine in the pathogenesis of T1R.

## 1. Introduction

Leprosy is a chronic infectious disease caused by the intracellular pathogen *Mycobacterium leprae* and that exhibits different clinical presentations. The clinical forms of the disease depend on the host’s immunological profile, ranging from a paucibacillary (PB) pole, characterized by the presence of a cellular immune response against the mycobacterial antigens and lower bacillary load, to a multibacillary (MB) pole, characterized by a low or absent cellular immune response against the mycobacterial antigens and higher bacillary load. During the clinical course of the disease, 30–50% of cases can present acute inflammatory episodes that may be classified as two main types: type 1 or reversal reaction (T1R), and type 2-reaction or erythema nodosum leprosum [1]. T1R involves exacerbation of old lesions and can be the first sign of the disease, persisting for a few weeks or months. T1R may occur in leprosy patients before, during, or after multidrug therapy (MDT) [2,3], and may be associated with an increase in neural damage. This damage is frequently connected to sequelae or deformities [4,5,6] and represents a challenge for clinical management [7,8]. T1R is treated with oral corticosteroids over a 4-to-6-month period, once specific drugs to treat T1R are not available [9,10,11,12]. Early diagnosis and the occurrence of T1R after completion of MDT are major challenges for T1R management. There is an urgent need for the identification of molecular, immunological, and genetic mechanisms that might aid in early recognition and the administration of adequate treatments to prevent T1R complications.

T1R occurs mainly in the unstable borderline forms of leprosy (borderline tuberculoid (BT), borderline borderline (BB), and borderline lepromatous (BL)) and subpolar lepromatous lepromatous (LL) forms [2,3,4]. Our previous results have demonstrated that skin cells from MB patients have a blockade in the autophagy flux, which in turn contributes to the increase in the bacillary load [13]. Mycobacteria can manipulate intracellular signaling pathways to escape from host-defense mechanisms in human cells. Autophagy is a crucial mechanism during mycobacterial infections, and during *M. leprae* infection, a blockade in Beclin 1-mediated autophagy may contribute to the pathogenesis of MB leprosy [13]. However, some MB patients may develop T1R during the clinical course of the disease, and, as previously observed, in these cases, autophagy is restored by a mechanism that involves the pro-inflammatory cytokine interferon-gamma (IFN-*γ*) [13].

T1R is associated with a cellular immune response to mycobacterial antigens. It has been demonstrated that cytokines derived from Th1 cells, such as interleukin (IL)-1*β*, tumor necrosis factor (TNF), IL-2, and IFN-*γ*, play important roles in the pathogenesis of T1R [14]. A cross-sectional study has demonstrated that five genes are associated with the outcome of T1R in leprosy patients: *CCL2*, *CD8A*, *IL2*, *IL15*, and *MARCO* [15]. In addition, intra-individual longitudinal analyses demonstrated that several IFN-*γ*-induced genes increased significantly at the onset of T1R, whereas IL-15 decreased [15].

The comprehension of T1R-related mechanisms is limited, mainly because studies have evaluated the immune response during the acute inflammatory episode [16,17], whereas several inflammatory mediators are increased. On the basis of previous studies, we found that it is not possible to establish the initial immunopathogenic effectors responsible for T1R onset, nor the contribution of each molecule. Previous studies have shown that MB skin macrophages predominantly exhibit an anti-inflammatory phenotype and functional role with increased expression of IFN-*β* [18,19,20]. During T1R, however, the elevated systemic IFN-*γ* production [21] may contribute to the co-existence of mixed pro- and anti-inflammatory phenotypes. Because no experimental model exists to study leprosy, or to evaluate T1R, we attempted to investigate whether differentiated THP-1 macrophages stimulated with *M. leprae* in the presence of IFN-*γ* could mimic the process observed in skin tissue/cells during T1R, while also assessing the impact of IFN-*γ* treatment on autophagy modulation. We relied on the commonly used autophagic marker LC3 (microtubule-associated protein 1 light chain 3) as an indicator of autophagy activity [22]. The pro-LC3 protein is first cleaved post-translationally into the cytoplasmatic soluble form LC3-I. After processing, LC3-I is conjugated to phosphatidylethanolamine (PE) by the Atg3 protein, generating the membrane-associated LC3-II (also known as LC3-PE), which correlates with the extent of autophagosomes [22].

Our previous study indicated that MB patients that develop T1R during treatment exhibit an autophagy pathway blockade in skin cells, which results in increased inflammasome activation [23]. In this study, our results show that patients who progress to T1R had increased levels of IL-15 even before the beginning of the reaction, leading us to hypothesize that IL-15 binds to the IL-15R complex on CD4 T cells and contributes to IFN-*γ* production. Once established, IL-15 production is reduced and IFN-*γ* acts on host cells by inducing autophagy, corroborating recently published findings [15].

Our results also demonstrate that IFN-*γ* induced autophagic flux and increased IL-15 secretion in THP-1 macrophages stimulated with *M. leprae*. Blocking the autophagic pathway with 3-methyladenine (3-MA) led to a reduction in IL-15 levels but did not increase IL-10 production, suggesting that IL-15 synthesis depends on IFN-*γ*-mediated autophagy. Gene expression analysis in THP-1 cells stimulated with *M. leprae* in the presence of IFN-*γ* showed a positive regulation of key autophagy genes (*RPTOR*, *SEC23B*, *LAMP2*, *ATG7*, *ATG10*, *ATG16L2*, *LAMP2*, *ULK2*, *SQSTM1*, *FKBP15*) when compared to *M. leprae* alone. An analysis of overlapping gene signatures revealed the presence of 13 common genes (17.6%) between T1R skin lesions and THP-1 cells treated with both *M. leprae* and IFN-*γ*, including the autophagy modulator *TPR*. Overall, these data suggest that IFN-*γ* modulates the autophagy pathway during T1R, and stimulation of differentiated THP-1 cells with IFN-*γ* can modulate the expression of autophagy genes that are upregulated in skin cells from T1R patients.

## 2. Materials and Methods

### 2.1. Patients and Clinical Specimens

Leprosy patients involved in the study were recruited from Souza Araújo Outpatient Unit (FIOCRUZ) and classified accordingly to the criteria of Ridley and Jopling [24]. Volunteers (*n* = 41) were included in the study, including 14 paucibacillary patients (PB), 15 multibacillary patients (MB), and 12 patients with T1R. As observed in Table 1, among the 15 MB patients included in the study, 7 were diagnosed with T1R during the clinical follow-up. The skin biopsies of PB and MB patients were taken at the time of leprosy diagnosis and, thus, prior to MDT. T1R biopsies were obtained during T1R episodes in patients originally diagnosed with BL or subpolar LL clinical forms. The clinical and demographic data of all patients recruited in this study are presented in Table 1.

### 2.2. Ethics Statement

This study was approved by the FIOCRUZ Institutional Ethics Committee (approval number 1.538.467, approval date: 10 May 2016). Written informed consent was obtained from all patients before inclusion in the study.

### 2.3. Cell Culture

The human monocytic cell line THP-1 (ATCC TIB-202) was grown in suspension as described elsewhere [13]. For the differentiation of THP-1 monocytes into macrophages, we suspended cells in complete medium containing 200 nM phorbol 12-myristate 13-acetate (PMA; Sigma-Aldrich, St. Louis, MO, USA) for 24 h at 37 °C. After this period, non-adherent cells were removed by washing with phosphate-buffered saline (PBS; Sigma-Aldrich) at 37 °C and resuspended in RPMI 1640 supplemented with 10% FBS, 2 mM L-alanyl-L-glutamine, and 100 U/mL penicillin and 100 μg/mL streptomycin (GIBCO, Waltham, MA, USA). For primary monocyte isolation, peripheral blood mononuclear cells (PBMCs) from healthy volunteers were isolated under endotoxin-free conditions by density sedimentation over Ficoll–Paque gradient (GE Healthcare Life Science, Little Chalfont, United Kingdom). PBMCs were allowed to adhere to culture plates for 2 h at 37 °C, then non-adherent cells were removed by vigorous washing with PBS.

### 2.4. Mycobacteria

*M. leprae* Thai-53 isolate was grown in the footpad of athymic nude mice as described elsewhere [25] and was provided by the National Hansen’s Disease Program. To obtain a fluorescent *M*. *leprae*, we labeled the bacterial suspensions with PKH26 Red Fluorescent Cell Linker Kit (Sigma-Aldrich) according to the manufacturer’s instructions. THP-1 macrophages and primary human monocytes were stimulated with irradiated (dead) PKH-labeled *M*. *leprae* at a multiplicity of infection (MOI) of 10:1 organisms/cell for 30 min before autophagy induction and then incubated for 18 h at 37 °C in 5% CO_2_.

### 2.5. Autophagy

Autophagy induction was performed via treatment with recombinant human IFN-*γ* (10 ng/mL; BD PharMingen, San Diego, NJ, USA) or rapamycin (200 ng/mL; Enzo Life Sciences, Farmingdale, NY, USA) for 18 h at 37 °C in 5% CO_2_. When indicated, autophagy inhibition was performed using the pharmacological inhibitors Wortmannin (Wtm) (100 nM; Enzo Life Sciences) or 3-methyladenine (3-MA) (10 mM; Sigma-Aldrich) for 1 h before induction of autophagy. Autophagy induction was assessed by Western blotting, fluorescence microscopy, and transmission electron microscopy.

### 2.6. Transmission Electron Microscopy (TEM)

Adherent monocytes were cultured in 25 cm^2^ cell culture flasks (Corning, NY, USA) and fixed with 2.5% glutaraldehyde in 0.2 M cacodylate buffer pH 7.2 for 24 h at 4 °C, and postfixed in 2% osmium tetroxide in 0.1 M cacodylate buffer for 1 h at 4 °C. Then, cells were detached using a cell scraper, transferred to an Eppendorf tube, and dehydrated in graded acetone series. Next, cells were pelleted by centrifugation (300× *g*, 10 min, 25 °C) and the acetone was removed and replaced by Epoxy resin (Embed-812 kit). Finally, a small sample piece was transferred to an embedding mold and polymerized at 60 °C for 72 h to generate the final resin block. Ultrathin 70 ± 80 nm sections were collected on 300-mesh copper grids, stained with uranyl acetate and 2% lead citrate, and viewed using a JEOL JEM-1011 transmission electron microscope (JEOL, Tokyo, Japan) operating at 60 kV beam voltage. All the reagents were purchased from Electron Microscopy Sciences unless otherwise specified. Images were digitally captured with a SC1000 ORIUS CCD camera (Gatan Inc., Pleasanton, CA, USA) using Gatan Digital Micrograph 2.31.734.0 software (Gatan Inc.).

### 2.7. Immunofluorescence Staining

Cells were cultivated on 15 mm sterile circular coverslips placed on the bottom of 24-well microplates (Corning). Monolayers were fixed with 4% paraformaldehyde (Sigma-Aldrich) at 25 °C for 20 min. Cells were then washed three times with 0.05% saponin (Sigma-Aldrich) in PBS, blocked with 10% normal goat serum (NGS; Sigma-Aldrich) for 1 h at room temperature, and probed with the primary antibodies—mouse IgG1 anti-human microtubule associated protein 1 light chain 3 (LC3) (1:50; MBL International, Sunnyvale, CA, USA, M152-3) or mouse IgG_2b_ anti-human autophagy-related protein 3 (Atg3) (1:300; Santa Cruz Biotechnology sc-393660 A-3, Dallas, TX, USA)—overnight at 4 °C. Then, cells were washed and incubated with the secondary antibodies Alexa Fluor 633 goat anti-mouse IgG1 (1:500; Molecular Probes, Eugene, OR, USA, A21126) or Alexa Fluor 488 goat anti-mouse IgG_2b_ (1:500; Molecular Probes, A21141), respectively, for 2 h at room temperature. For lysosome staining, the acidotropic dye LysoTracker Yellow HCK-123 (500 nM; Molecular Probes) was added to the cultures 30 min before fixation. Finally, nuclei were stained with 4′-6-diamidino-2-phenylindole (DAPI; 1:10,000, Molecular Probes). The coverslips were mounted with PermaFluor Aqueous Medium (Thermo Scientific, Waltham, MA, USA).

Cells were imaged using an Axio Observer.Z1 microscope equipped with Colibri.2 and ApoTome.2 illumination systems (Carl Zeiss, Oberkochen, Germany) and the EC Plan-Neofluar 40×/1.30, 63×/1.40, and 100×/1.30 oil objectives. Images were acquired with an AxioCam HRm digital camera and AxioVision Rel. 4.8.2.0 software (Carl Zeiss). The number of fluorescent LC3 puncta (3 µm^2^) was quantified using the Particle Analyzer plugin of the ImageJ software after image thresholding, and the image calculator tool was used to measure Atg3 fluorescence intensity (mean gray value) [26,27]. ImageJ was also used for colocalization analysis. Briefly, the red, green, and blue channels were extracted from RGB images and converted to binary images (grayscale) by automatic thresholding. Next, the background pixilation was removed from the analysis, and then merged channels were analyzed using the Colocalization and Analyze Particles built-in functions of the software [27]. For both analyses, a minimum of 100 cells per sample was scored for each experiment.

### 2.8. Cytokine Measurement

Cytokine levels in cell culture supernatants were measured either by ELISA (R&D Systems, Minneapolis, MN, USA) or by Bio-Plex Multiplex Immunoassays (Bio-Rad Laboratories, Hercules, CA, USA), according to the manufacturer’s instructions. Absorbances were measured using the SpectraMax 190 microplate reader and analyzed using the SoftMax Pro v5.3 software (Molecular Devices, Silicon Valley, CA, USA). Fluorescence levels were determined using a Luminex-based Bio-Plex Multiplex Reader System (Bio-Rad Laboratories).

### 2.9. RNA Isolation, Reverse Transcription, and Real-Time PCR Array Quantifications

RNA was extracted from THP-1 macrophages by TRIzol reagent (ThermoFisher Scientific), following the manufacturer’s instructions. For skin lesions, 4 mm punches were surgically excised and transferred to 1 mL of RNA later stabilization solution (ThermoFisher Scientific). Total RNA was isolated using Polytron Homogenizer Model PT3100 apparatus (Kinematica AG, Lucerne, Switzerland) in 2 mL of TRIzol. After isolation, RNA samples were treated with the TURBO Dnase (ThermoFisher Scientific) and checked for integrity and purity by 1.2% agarose gel electrophoresis. One microgram of total Dnase-treated RNA was reverse transcribed into complementary DNA using SuperScript VILO Master Mix, according to the manufacturer’s instructions (ThermoFisher Scientific). Real-time gene expression (RT-qPCR) was performed with a human autophagy PCR array (Real Time Primers, Elkins Park, PA, USA, HATPL-I) composed of 88 autophagy-associated targets and 8 reference genes. The full list of genes is available on http://realtimeprimers.com/huauprli.html (accessed on 1 May 2021). The RT-qPCR autophagy array was conducted with Power SYBR Green PCR Master Mix (Applied Biosystems, Beverly, MA, USA) and PCR conditions according to the manufacturer’s recommendations. RT-qPCR reactions were cycled in a ViiA 7 Real-Time PCR System and analyzed with QuantStudio V1.2.4 and ExpressionSuite V1.1 software (Applied Biosystems, Beverly, MA, USA). Gene expression data were analyzed by the 2^−ΔΔCT^ method and normalized using the reference gene hypoxanthine phosphoribosyltransferase 1 (*HPRT1*). In this study, the *HPRT1* gene was the best endogenous control due to the lowest score variation value (determined by ExpressionSuite V1.1) and as determined in our previous studies using a qPCR array [13,23].

### 2.10. Gene Expression Analysis of IL-15 and IL-10 by Real-Time RT-qPCR

Quantitative RT-qPCR was carried out in a total reaction volume of 10 µL containing 1× each of TaqMan gene expression assays (20×) (human *IL10* (Hs00174086_m1), and human *IL15* (Hs01003716_m1), ThermoFisher Scientific), 1× TaqMan Fast Advanced Master Mix (2×) (ThermoFisher Scientific), and 10 ng of cDNA. RT-qPCR reactions were performed in a StepOnePlus Real-Time PCR Systems thermocycler (ThermoFisher Scientific), and relative expression of target genes was normalized by human *GAPDH* (Hs99999905_m1; Thermo Fisher Scientific), a very common reference gene used in the literature and highly standardized in our laboratory [13,23]. RT-qPCR data analysis was performed with the N_0_ method implemented in LinRegPCR v. 2020.0, which considers RT-qPCR mean efficiencies estimated by the window-of-linearity method [28,29]. Briefly, N_0_ values were calculated in LinRegPCR using default parameters. Then, N_0_ values from the gene of interest (GOI) were normalized by taking their ratio to the N_0_ of the reference gene *GAPDH* (N_0GOI_/N_0REF_).

### 2.11. Autophagy Pathway Analysis

Gene expression profiles of THP-1 macrophages were derived from the RT-qPCR autophagy array. Genes that were expressed differentially in the two groups were identified by fold change ≥1.5-fold thresholds [18,30]. Heat maps were generated using the Enhanced Heat Map (heatmap.2) function from the “gplots” R package and displayed after z-score scaling. Autophagy-associated genes displayed on heat maps were functionally sub-categorized into nine subgroups according to a previously published list of human gene symbols involved in autophagy and lysosomal pathways [31]. A Venn diagram was generated using VENNY 2.1 [32]. Autophagy gene signatures of leprosy lesions were obtained from reference [13] and are available on Table S1. The Search Tool for the Retrieval of Interacting Genes/Proteins (STRING version 11.0) database (http://string-db.org/) was used for protein interaction analysis (accessed on 5 June 2021) [33]. Network maps of protein–protein interactions were obtained through the evidence view on STRING.

### 2.12. Leprosy Skin Biopsy Gene Expression from Public Microarray Data

Raw data from Belone and colleagues (GSE74481) [34] were downloaded from Gene Expression Omnibus (GEO). Raw data were imported into the R environment v. 3.4.1 with the help of the Biobase v. 2.36.2 and limma v. 3.32.6 packages. Unprocessed intensity values were background-corrected and normalized using Robust Multi-array Average (RMA) and quantile normalization, respectively. Duplicated probes/genes were removed by keeping the one with the greatest mean across all samples. Normalized log_2_ values were used for data visualization using ggplot2 v. 3.0.0. The a priori list of genes to be screened in null hypothesis testing was as follows: *TPR*, *GFI1B*, *GNAI3*, *GPSM1*, *GPSM2*, *LETM2*, *BECN2*, *RASD1*, *RPTOR*, *FRS3*, *UVRAG*, *SEC24A*, and *SEC23B. BECN2* was excluded because of microarray annotation issues. Null hypothesis testing was undertaken using ANOVA for non-constant variance by fitting linear models using generalized least squares (nlme::gls, v. 3.1-148). Pairwise comparisons among the MB (LL + BL), PB (TT + BT), and T1R groups were tested using the Tukey procedure as implemented by the emmeans v. 1.4.4 package. Null hypotheses were rejected if Tukey *p*-values were smaller than or equal to 0.1.

### 2.13. Western Blot

Western blotting was carried out as previously described [13,23]. Briefly, equivalent amounts of protein (20 μg) from THP-1 cells were resolved on 12% polyacrylamide gels by SDS-PAGE. Resolved proteins were transferred to Hybond-C Extra nitrocellulose membranes (Amersham Biosciences, NJ, USA) in 25 mM Tris (Bio-Rad, CA, USA), 190 mM glycine (Bio-Rad), and 20% methanol (Merck, New Jersey, USA) using an electrophoretic transfer system with cold-block (Bio-Rad). The membranes were blocked using 5% bovine serum albumin (BSA; Sigma-Aldrich) in TBS containing 0.1% Tween-20 (Calbiochem) at room temperature for 1 h. After blocking, the membranes were immunoblotted sequentially overnight with primary antibodies against LC3 (1:400; MBL International) or glyceraldehyde-3-phosphate dehydrogenase (GAPDH; 1:500; mouse IgG1 anti-human, Santa Cruz Biotechnology, sc-47724). Incubations were followed by washing, and bound antibodies were detected with the appropriate HRP-conjugated secondary antibody goat anti-mouse IgG-HRP (1:2000; DakoCytomation, Glostrup, Denmark P0447) for 1 h at room temperature. Immuno-reactive bands were detected using the chemoluminescent substrate Western blotting luminol reagent (Santa Cruz Biotechnology) and revealed using medical X-ray film (Carestream Kodak X-Omat LS film, Amersham Biosciences). Densitometric analysis was performed using Adobe Photoshop CS6 software (Adobe Systems Incorporated, San Jose, CA, USA).

### 2.14. Statistical Analysis

Statistical analysis and graphing were undertaken with GraphPad Prism 9 software (GraphPad, San Diego, CA, USA). Statistics reported are of entire series of experiments and described as mean ± the standard error, or whiskers indicating minimum to maximum with all points shown. For comparison between three or more groups with matched or repeated data, we used RM one-way or two-way ANOVA, with the Geisser–Greenhouse correction, in addition to Tukey’s multiple comparisons test, with individual variances computed for each comparison; for data without matching or pairing, we used Brown–Forsythe and Welch ANOVA tests with Dunnett’s T3 multiple comparisons test. For two-group analysis, a two-tailed paired Student’s *t*-test or a two-tailed unpaired *t*-test with Welch’s correction was used. A *p*-value < 0.05 was considered statistically significant.

## 3. Results

### 3.1. IFN-γ Induced the Formation of Autophagosomes in Human Macrophages Stimulated with M. leprae

**Figure 1:**
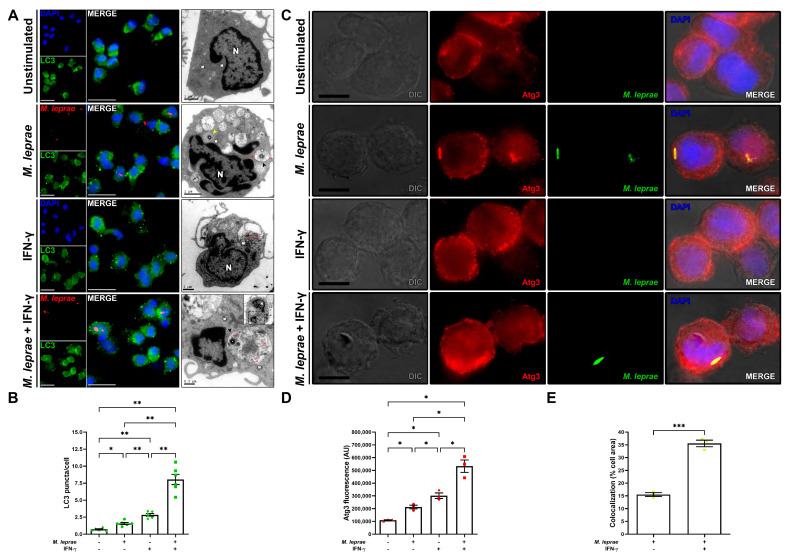
IFN-*γ* positively regulated autophagy in monocytes stimulated with *M. leprae*. (**A**) Human monocytes were stimulated with *M. leprae* (MOI 10:1) for 30 min, then treated with 10 ng/mL of IFN-*γ* for 18 h and processed for analysis by immunofluorescence or transmission electron microscopy. LC3 (green), *M. leprae* stained with PKH26 (red), and the nucleus stained with DAPI (blue). Red arrows indicate double-membrane (autophagosomes) visible sites; black arrowheads indicate *M. leprae*-containing autophagosomes; yellow arrowheads indicate *M. leprae*-containing phagosomes; asterisks indicate *M. leprae*; M indicates mitochondria; N indicates nucleus. The images are representative of six (fluorescence) or three (TEM) experiments. Scale bar: 20 µm. (**B**) Quantification of LC3 puncta per cell in the same conditions mentioned above. Data are the mean ± S.E.M; * *p* < 0.05, ** *p* < 0.01 by the repeated measures one-way ANOVA with the Geisser–Greenhouse correction and Tukey’s multiple comparisons test. (**C**) Human THP-1 macrophages were stimulated with *M. leprae*-PKH26 (green) for 30 min (MOI 10:1) and treated with 10 ng/mL of IFN-*γ* for 18 h. Atg3 expression was evaluated by immunofluorescence microscopy using anti-Atg3 antibody (red), with the nucleus stained with DAPI (blue). The images are representative of three experiments. Scale bar: 10 µm. Differential interference contrast (DIC). (**D**) Quantification of Atg3 fluorescence in the same conditions mentioned above. (**E**) Quantification of the percentage of *M. leprae* organisms colocalized with Atg3. Data are the mean ± S.E.M; * *p* < 0.05, *** *p* < 0.001 by the repeated measures one-way ANOVA with the Geisser–Greenhouse correction and Tukey’s multiple comparisons test (**D**) or two-tailed paired *t*-test (**E**).

IFN-*γ* is known to induce autophagy [13]. We sought to determine whether this cytokine activates autophagy in our cell model to study T1R. The expression of lipidated LC3-II (or LC3-PE) was evaluated by immunofluorescence (Figure 1A) and Western blotting (Figure S1). IFN-*γ* treatment of primary human monocytes previously stimulated with *M. leprae* increased the number of LC3-II puncta (a hallmark of autophagosome induction) relative to stimuli alone, or unstimulated controls (Figure 1A,B). Immunoblot analysis confirmed that the proportion of LC3 present in the lipidated LC3-II form was higher in THP-1 macrophages stimulated with *M. leprae* and IFN-*γ*, which was inhibited when the cells were incubated in the presence of the autophagy inhibitor 3-methyladenine (3-MA) (Figure S1). TEM analysis revealed the presence of either phagosomes or autophagosomes containing bacteria in monocytes stimulated with *M. leprae* but not in unstimulated cells (Figure 1A). IFN-*γ* stimuli alone promoted the formation of double-membrane vacuoles (Figure 1A). In cells stimulated with both *M. leprae* and IFN-*γ*, autophagosomes harboring *M. leprae* were preferably detected (Figure 1A), confirming that the LC3 punctate staining observed in Figure 1A was due to lipidation of autophagosomes. Furthermore, we observed onion/myelin-like multilamellar structures, which are a hallmark of mycobacteria encapsulation by autophagy, and electron-lucent structures resembling damaged bacilli in close apposition to mitochondria and endoplasmic reticulum (sites of phagophore initiation), only in THP-1 macrophages that received both *M. leprae* and IFN-*γ* treatments (Figure S2). We next evaluated the expression of Atg3, an enzyme responsible for the conjugation of LC3-I to PE, forming LC3-II [35]. Compared to unstimulated cells or *M. leprae* alone, stimulation with IFN-*γ* increased Atg3 expression in human THP-1 macrophages (Figure 1C,D). We noticed that in IFN-*γ*-stimulated macrophages, Atg3 expression concentrated at the plasma membranes, which is a site of bacterial entry and autophagosome formation [36]. In addition, in the presence of IFN-*γ*, there was an increase in the colocalization of Atg3 and *M. leprae* (Figure 1C,E).

### 3.2. IFN-γ Treatment Induced Autophagy Flux in THP-1 Macrophages Stimulated with M. leprae

**Figure 2:**
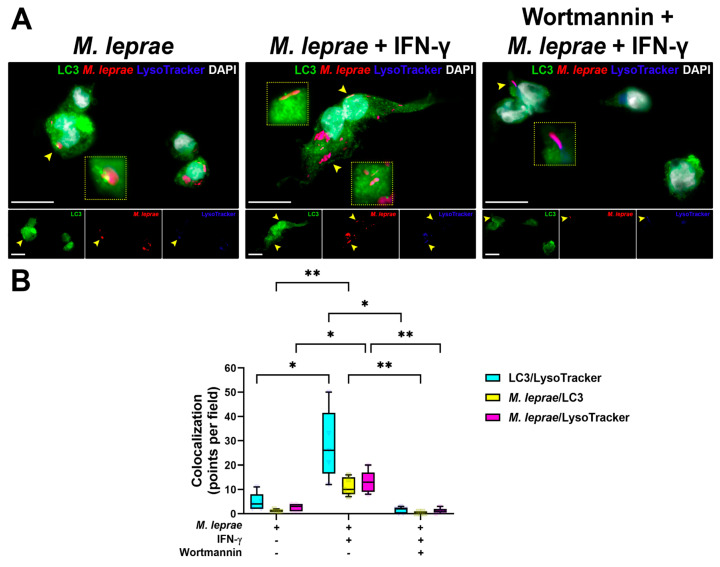
Wortmannin blockaded the autophagic flux induced by IFN-*γ* in THP-1 macrophages stimulated with *M. leprae.* (**A**) THP-1 macrophages were pretreated with 100 nM wortmannin for1 h, stimulated with *M. leprae* PKH26 (red) for 30 min (MOI 10:1), and treated with 10 ng/mL IFN-*γ* for 18 h. LC3 expression was assessed by immunofluorescence using an anti-LC3 antibody (green), with the nucleus stained with DAPI (white). LysoTracker (blue) was added to the cultures 30 min before fixation. Arrowheads indicate colocalization profiles in at least two channels, which are shown on the insets. Scale bar: 20 µm. (**B**) Colocalization analysis. The images represent five experiments. Data in whiskers are the minimum to maximum with all points shown; * *p* < 0.05, ** *p* < 0.01 by the repeated measures two-way ANOVA with the Geisser-Greenhouse correction and Tukey’s multiple comparisons test.

The last step of the autophagic pathway is the maturation of autophagosomes into degradative compartments called autolysosomes through the fusion with lysosomes, a process known as autophagy flux. We next investigated the IFN-*γ* effects on autophagosome maturation. Analysis of autophagy flux demonstrated that LC3-II punctate structures were observed in THP-1 macrophages stimulated with *M. leprae* alone, some of which were in juxtaposition/colocalization with lysosomes as stained by LysoTracker (Figure 2A). More significantly, induction of autophagy with IFN-*γ* was able to promote the colocalization of LC3-II-decorated autophagosomes with LysoTracker-positive lysosomes and *M. leprae* (Figure 2A). Pretreatment with the phosphatidylinositol 3-kinase (PI3K) inhibitor wortmannin was able to inhibit LC3-II expression induced by IFN-*γ* + *M. leprae* in THP-1 macrophages (Figure 2A). By quantifying each autophagy compartment by colocalization analysis, we observed that THP-1 cells treated with IFN-*γ* and *M. leprae* were prominently stained for autolysosomes and had more mycobacteria labeled for autophagosomes and lysosomes in comparison to *M. leprae* alone or in the presence of wortmannin (Figure 2B).

### 3.3. IFN-γ Upregulated Autophagy Gene Expression in M. leprae-Stimulated THP-1 Cells

**Figure 3:**
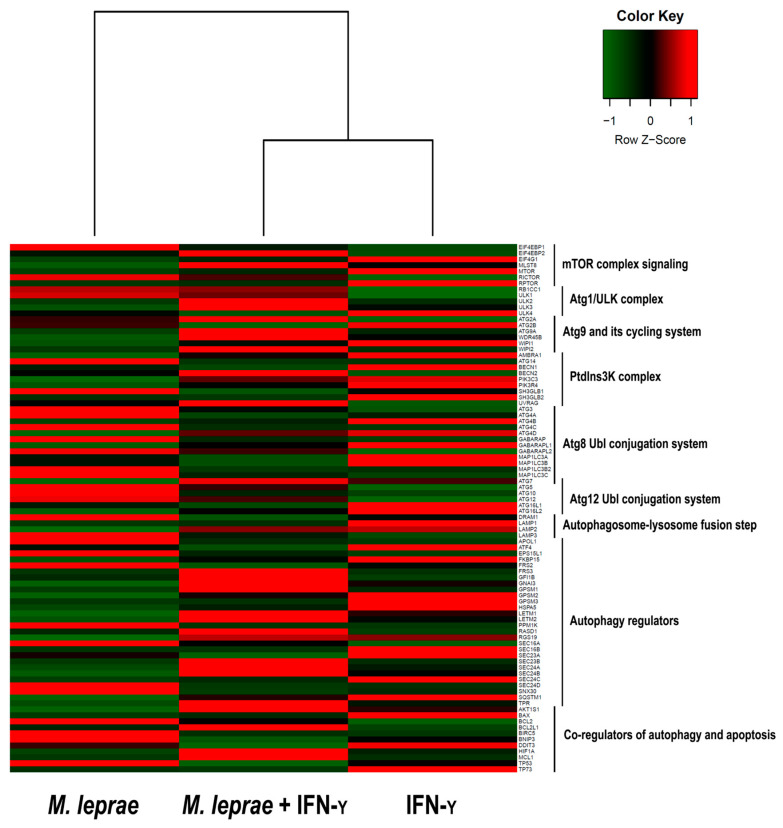
Autophagy gene expression profiles of THP-1 cells stimulated with *M. leprae* and IFN-*γ*. mRNAs of THP-1 macrophages treated with *M. leprae* + IFN-*γ*, as well as the macrophages treated individually with each stimulus, were analyzed by RT-qPCR using an autophagy pathway array PCR kit. The heatmap shows the analysis of autophagy-related genes aggregated in different categories. Each line is representative of a gene. Data represent three independent experiments.

To identify the components of the autophagic machinery involved in the cellular response to mycobacterial infection, we examined the expression of 88 genes related to autophagy. As shown in Figure 3, IFN-*γ* induced a positive regulation of genes related to the autophagy process in cultures stimulated with *M. leprae* (Table S1). Genes involved in the formation of autophagosomes (*ULK2*, *ATG16L2*, *ATG10*, and *ATG7*), autophagy regulation (*FKBP15*, *GPSM1*, *GPSM2*, *SEC23B*, and *SQSTM1*), and lysosomal pathways (*LAMP2*) were upregulated by IFN-*γ* in *M. leprae*-stimulated THP-1 cells (Figure 3).

### 3.4. IFN-γ Increased IL-15 Secretion in THP-1 Macrophages Stimulated with M. leprae

**Figure 4:**
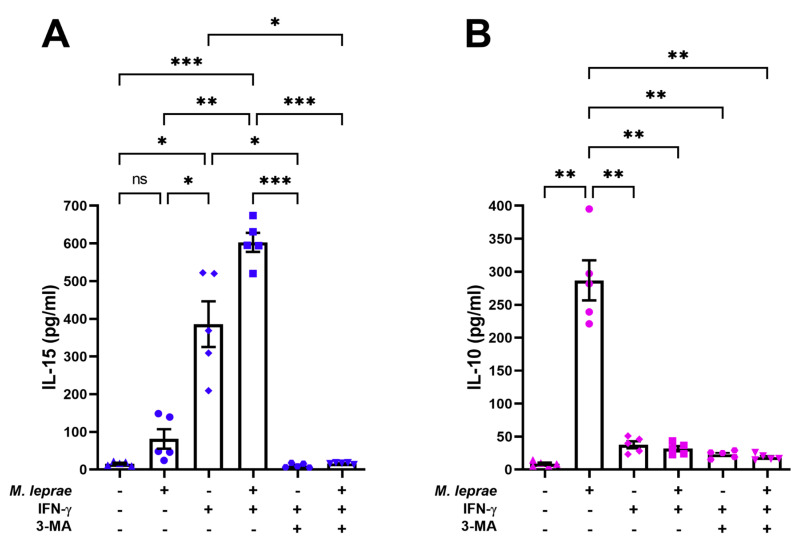
In the presence of *M. leprae*, IFN-*γ* increased the secretion of IL-15 in THP-1 macrophages. THP-1 macrophages were pretreated or not with 10 mM 3-MA for 1 h, stimulated with *M. leprae* for 30 min (MOI 10:1), and treated with 10 ng/mL of IFN-*γ* for 18 h. Cytokine production was assessed by ELISA. (**A**) IL-15, (**B**) IL-10. The graphs are representative of five experiments. Data are the mean ± S.E.M; * *p* < 0.05, ** *p* < 0.01, *** *p* < 0.001 by the repeated measures one-way ANOVA with the Geisser-Greenhouse correction and Tukey’s multiple comparisons test. ns: not significant.

Because of the increased levels of IL-15 in patients who develop T1R [15], we sought to evaluate whether the cytokine levels are altered in macrophages stimulated with IFN-*γ* in the presence or absence of *M. leprae*. The treatment of macrophages with IFN-*γ* was able to significantly increase the secretion of the pro-inflammatory cytokines IL-6, IL-12p40, TNF (not shown), and IL-15, but not IL-10, relative to unstimulated cultures (Figure 4A,B). In the presence of both *M. leprae* and IFN-*γ*, there was an increase in IL-15 and a decrease in IL-10 secretion, compared to *M. leprae* alone (Figure 4A,B). The increase in IL-15 in cultures stimulated with IFN-*γ* or *M. leprae* + IFN-*γ* was totally inhibited in the presence of 3-MA (Figure 4A). In contrast, autophagy blocking did not restore the IL-10 levels (Figure 4B). These results suggest that the induction of IL-15 by IFN-*γ* might be associated with autophagy.

### 3.5. Proinflammatory Cytokine IL-15 Was Increased in MB Skin Lesions That Progressed to T1R

**Figure 5:**
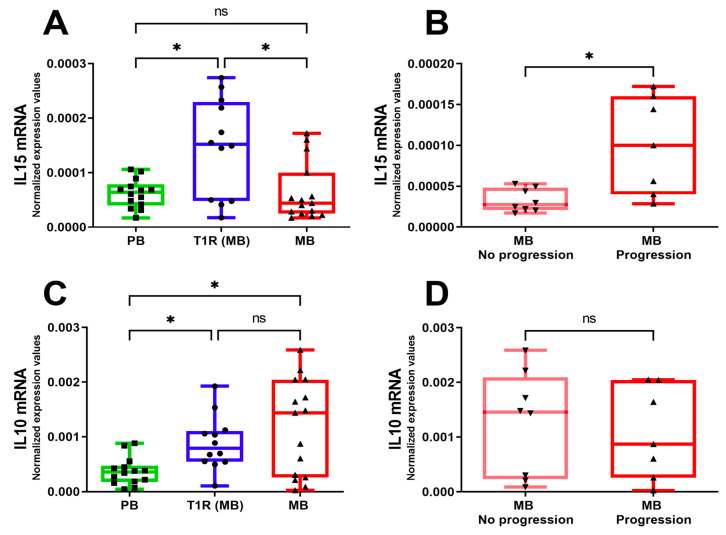
Cytokine mRNA expression profile in PB, MB, and T1R skin lesions. Gene expression of the inflammatory molecule IL-15 (**A**) and anti-inflammatory molecule IL-10 (**C**) were evaluated by real-time PCR in skin lesions of paucibacillary (PB), multibacillary (MB), and reactional (T1R) patients. (**B**,**D**) MB patients were monitored for 2 years following the start of MDT and were stratified on the basis of the outcome or not of T1R during the follow-up. (**B**) IL-15 mRNA levels. (**D**) IL-10 mRNA levels. Patient data are representative of PB (*n* = 14), MB no-progression (*n* = 8), MB progression (*n* = 7), and T1R (*n* = 12). Gene expression data were normalized to *GAPDH*. Data in the whiskers are the minimum to maximum with all points shown; * *p* < 0.05 by the Brown–Forsythe and Welch ANOVA tests with Dunnett’s T3 multiple comparisons test (**A**,**C**) or two-tailed unpaired *t*-test with Welch’s correction (**B**,**D**). ns: not significant.

Because IL-15 was modulated by IFN-*γ* in THP-1 cells stimulated with *M. leprae,* we evaluated the expression of both *IL10* and *IL15* in skin lesion cells from MB patients that either developed or did not develop T1R during the clinical follow-up. Increased expression of *IL15* was observed in T1R lesions compared to PB and MB groups (Figure 5A). Furthermore, we monitored the MB patients for 2 years for the development of T1R. Gene expression analysis showed differential regulation of *IL15* in samples from patients who developed T1R (MB progression) compared to those who did not progress to T1R (MB no progression) during the 2-year follow-up (Figure 5B). Conversely, T1R lesions exhibited increased expression of *IL10* (Figure 5C), whereas no differences were found in *IL10* expression levels between MB no-progression and MB progression skin lesions (Figure 5D).

### 3.6. Modulation of Autophagy Pathway in T1R Lesions Was Mediated by IFN-γ

**Figure 6:**
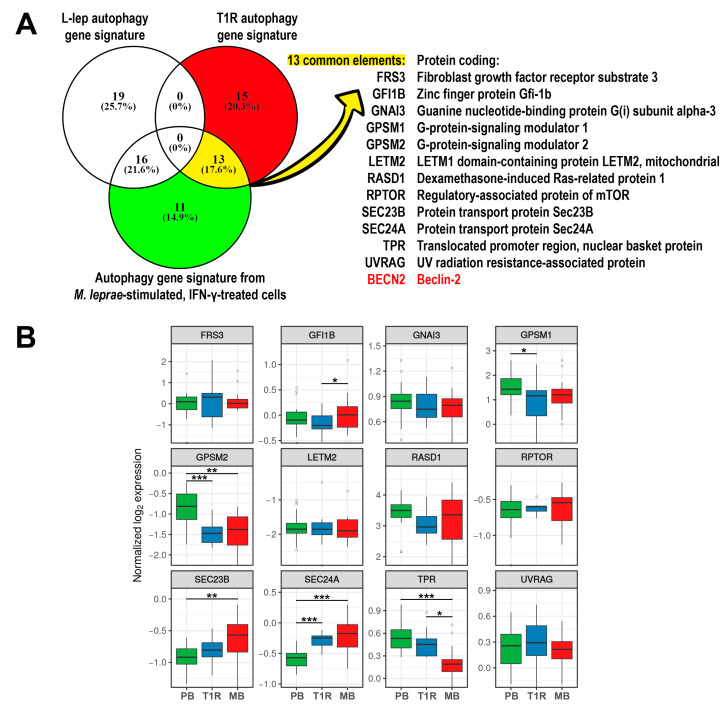
T1R skin lesions and THP-1 macrophages stimulated with *M. leprae* and IFN-*γ* were found to share a common autophagy gene signature. (**A**) Venn diagram overlap of autophagy gene expression signatures of leprosy skin lesions and THP-1 macrophages stimulated with *M. leprae* and treated IFN-*γ*. (**B**) Tukey box plots displaying normalized log_2_ gene expression values from microarray data [34] for the 13 genes found in (**A**). *BECN2* was excluded because of microarray annotation issues. Boxes represent first, second (median), and third quartiles with whiskers extending ± 1.5× the interquartile range (IQR). Means were compared using a linear mixed-effects model, allowing for heterogeneous variance among groups, followed by the Tukey multiple comparison procedure. Asterisks summarize Tukey *p*-values as * *p* < 0.05, ** *p* < 0.01, and *** *p* < 0.001. Tukey confidence intervals are described within results.

To establish a parallel between *M. leprae*-stimulated cell cultures incubated with IFN-*γ* and T1R skin lesion cells, we performed an intersection analysis (as illustrated by a Venn diagram) between an autophagy gene expression signature derived from THP-1 macrophages treated with both IFN-*γ* and *M. leprae* (Table S1 and Figure 3), and our previously published [13] autophagy signatures obtained from MB and T1R skin lesions (Table S1 and Figure S3). We observed the presence of 13 common genes (17.6%) between T1R skin lesions and *M. leprae* + IFN-*γ*-stimulated cells (*FRS3*, *GFI1B*, *GNAI3*, *GPSM1*, *GPSM2*, *LETM2*, *RASD1*, *RPTOR*, *SEC23B*, *SEC24A*, *TPR*, *UVRAG*, and *BECN2*) (Figure 6A). Re-analysis of leprosy skin biopsy gene expression from microarray data [33] showed that *TPR* expression was significantly increased in T1R vs. MB (log_2_FC 0.20, 95% Tukey CI [0.01–0.40]) and in PB vs. MB (log_2_FC 0.34, [0.16–0.51]). However, *SEC24A* was only upregulated in T1R compared to PB (log_2_FC 0.33, [0.14–0.52]). *GPSM1* and *GPSM2* genes were both downregulated in T1R compared to PB skin lesions (log_2_FC -0.67 [−1.29 to −0.04]; log_2_FC −0.60 [−0.95 to −0.25], respectively). *SEC24A* and *SEC23B* genes were upregulated in MB compared with PB lesions (log_2_FC 0.34 [0.15–0.54] and log_2_FC 0.31 [0.1–0.52], respectively). *GFI1B* had a higher mean expression in MB compared to T1R (log_2_FC 0.27, [0.02–0.56]) (Figure 6B).

**Figure 7:**
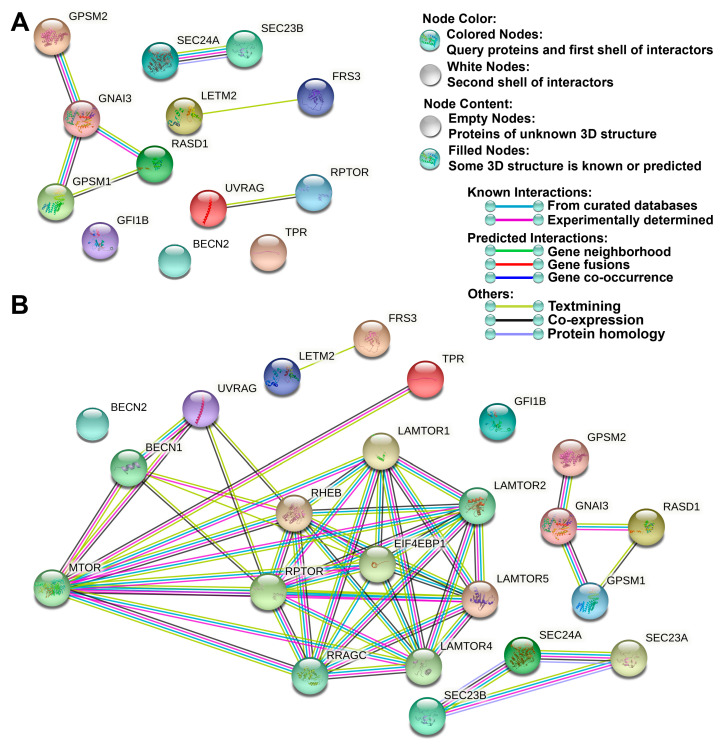
Autophagy protein interaction network between T1R skin lesions and THP-1 macrophages stimulated with *M. leprae* and IFN-*γ*. The 13 autophagy proteins codified by the upregulated genes in both T1R patients and treated THP-1 cells were visualized by STRING. (**A**) The evidence network view showing the 13 shared targets found in this study. In this view, colored lines between proteins indicate the various types of interactions. Network nodes represent proteins. Edges represent protein–protein associations. (**B**) More proteins were added to the evidence network shown in (**A**) to show the interaction of the 13 shared targets with core autophagy machinery proteins.

To better understand the relationship among the genes overlapped in THP-1 cells and T1R lesions, we further submitted the 13 common targets to protein–protein interaction analysis using the STRING database. Network maps of 13 common elements revealed three distinct protein interaction nodes: GNAI3, GPSM1, GPSM2, and RASD1; RPTOR and UVRAG; and SEC23B and SEC24A; furthermore, some protein–protein associations within these nodes were revealed (Figure 7A). By connecting the 13 common elements to core autophagic machinery proteins, we determined that TPR directly interacts with the central autophagy regulator mTOR, which in turn connects to RPTOR and UVRAG (Figure 7B).

## 4. Discussion

T1R is a model of a human pathological condition concerned with a chronic infectious disease and the instability of the immune response [23]. T1R is described as a condition associated with a reactivation of a previously suppressed specific immune response against mycobacteria in a scenario that is similar to an immune reconstitution syndrome, such as in HIV highly active anti-retroviral therapy [23]. Knowledge of the mechanisms related to the onset of T1R is pivotal for developing effective control strategies. These episodes are associated with an increment in neural damage caused by *M. leprae*, which is responsible for incapacities and deformities related to leprosy pathology [6,7]. Here, we provide evidence that 13 genes related to T1R pathogenesis are also modulated in *M. leprae*-stimulated cells in the presence of IFN-*γ*, and that IL-15 is involved in the pathogenesis of T1R.

Previous studies have demonstrated biomarkers related to T1R [37,38,39], and there is a consensus regarding the role of IFN-*γ* in the pathogenesis of this episode [40,41]. One limitation in the understanding of the initial steps in T1R pathogenesis is due to the lack of an experimental leprosy model that reproduces not only the different disease clinical forms, but also its reactional episodes. Another limitation is that clinical evaluation indicates T1R outcomes do not present a gradual start; rather, they usually occur abruptly and are associated with an increase in the levels of pro-inflammatory cytokines such as IFN-*γ*, TNF, and IL-6, and chemokines such as IP-10/CXCL-10 and MCP-1 [20,42]. This inflammatory milieu in T1R is frequently associated with mycobacterial killing, and the antigenic spread has an important function in determining the progression of the reactional episode. On the basis of the essential role of IFN-*γ* during T1R, we decided to evaluate how this cytokine modulates macrophage function when infected with *M. leprae*. Previous studies from our group have shown that IFN-*γ* induces host cell autophagy and is associated with the mycobactericidal activity of the macrophage [13]. In addition, we found that autophagy pathway blockade is associated with T1R outcomes in multibacillary patients [23]. Here, we evaluated if there is a parallel between the autophagy induction in cultures from differentiated THP-1 cells stimulated with both IFN-*γ* and irradiated *M. leprae*, as well as the autophagy induction in skin cells from multibacillary patients that develop T1R during the clinical follow-up.

Analysis of autophagy induction revealed that IFN-*γ* increased the formation of autophagosomes in cells previously stimulated with *M. leprae*. IFN-*γ* increased both the expression of Atg3 and the autophagic flux in *M. leprae*-stimulated cells, which was reverted by wortmannin. IFN-*γ* treatment amplified the expression of genes associated with signaling pathways that regulate lipid/obesity metabolism (*RPTOR*) [43]; vesicle trafficking (*SEC23B*) [44]; expansion of vesicles (*ATG7*, *ATG10*, and *ATG16L2*); and protection, maintenance, and adhesion of lysosomes (*LAMP2*) [44] in *M. leprae*-stimulated cultures. In addition, it also culminated in higher expression of genes that are part of the central autophagic machinery, such as *ULK2*, one of the members of the ATG1/ULK complex, which regulates autophagosome assembly [31]. Other genes include *SQSTM1*, which functions as an adapter protein linking ubiquitinated proteins to LC3 [45], and *FKBP15*, which inhibits mTOR when complexed with rapamycin [46].

Previous works have shown that pro-inflammatory cytokines can augment autophagosome formation [47]. MB patients have a predominantly Th2 immune response, and cytokines such as IL-4, IL-10, and IL-13 are associated with impaired autophagy induction, contributing to the higher bacillary load in MB cells [13,20]. Montoya and colleagues [19] demonstrated a dichotomy in IL-15 and IL-10 activity in macrophages. Although IL-15 is induced by IFN-*γ* and induces a vitamin D3-dependent antimicrobial pathway in PB patients, IL-10 induces the phagocytic pathway that is observed in macrophages from MB patients [19]. Here, we observed that IFN-*γ* increased IL-15 levels in *M. leprae*-stimulated cells and that the expression of *IL15*, but not *IL10*, was increased in skin cells from MB patients that developed T1R during the follow-up. It suggests that before the reactional clinical signs, there were inflammatory changes in the skin microenvironment partially modulated by IL-15. The human skin is a homing organ for T cells, both in physiological and pathological conditions, and local signaling by IL-15 is required for the formation of long-lived memory cells [48]. T1R is characterized by an increase in T cells that suggests intravascular activation at the beginning of reactional episodes [49]. IL-15 has been involved in the promotion of proliferation and survival of circulating memory T cells, and previous studies have demonstrated that IL-15 strongly induces perforin and granzyme B expression in CD8^+^CD103^+^CD49a^+^ cells [50]. In leprosy–HIV co-infected patients, T1R is characterized by an increased expression of effector memory CD8^+^ T cells, which is associated with the increased severity of T1R in these co-infected patients [51]. Despite the relevant role of CD8^+^ T cells in T1R pathogenesis, in this study we focused on the participation of innate immune mechanisms related to the onset of T1R in MB leprosy patients. However, we must not ignore the fact that the IL-15-mediated recruitment of skin T cells may contribute to the local production of IFN-*γ*, thus contributing to a positive feedback loop that will result in IL-15 production by skin cells such as keratinocytes and skin macrophages.

Andrade and colleagues [21] described that the beginning of T1R dramatically alters the organization and morphology of the lesion in MB patients, leading to the appearance of new cell structures and cell populations, such as epithelioid cells, granulomas, and a significant phenotypic diversity of macrophages and dendritic cells. IFN-*γ* induces IL-15 via the Stat1-dependent pathway, leading to the conversion of vitamin D3 and activation of an antimicrobial pathway mediated by autophagy, promoting autolysosomal fusion and *Mycobacterium tuberculosis* elimination [52,53]. Here, we observed that the pharmacological blockade of the autophagy pathway led to a reduction in IL-15 levels but did not increase IL-10 production. IL-15, like other pro-inflammatory cytokines, may exert a paracrine and autocrine action on cells, stimulating autophagy, which in turn would promote cytokine production, providing positive feedback. Wang and colleagues [54] demonstrated that the IL-15 produced by dendritic cells in response to stress binds to the receptor (IL15R) on CD4^+^ T cells, and induces CD40L expression, T cell proliferation, and IFN-*γ* production. CD40L expression in T CD4^+^ cells then reactivate CD40 molecules in the dendritic cell, inducing their maturation and IL-15 expression, maintaining a feedback circuit [54]. It has already been described that reactive skin lesions have a higher infiltration of CD4^+^ lymphocytes, in addition to greater cellular immune reactivity against *M. leprae* antigens [55]. Thus, it is suggested that macrophages from MB patients’ skin lesions that progress to T1R become activated and initiate an immune response against *M. leprae* components, which leads to the rupture of the classical *M. leprae*-induced immunosuppression seen in MB cells early in the T1R episode.

Intersection analysis of the differentially expressed gene lists revealed the presence of 13 common genes (17.6%) between T1R skin lesions and *M. leprae* + IFN-*γ*-stimulated cells. Among these genes, we found two central components of COPII vesicles, *SEC23* and *SEC24*; both are involved in membrane traffic between ER and Golgi in yeast and animal cells [56]. Furthermore, it has been shown that the SEC23/SEC24 complex is recruited to the ER membrane by SAR1, a G protein, which also participates in the protein coating complex [57,58]. As a central component of COPII vesicles, SEC23 is not only involved in the process of transporting and secreting proteins in cells but also participates in autophagy and promotes cancer cell survival [59]. The roles of COPII in the autophagic pathway are conserved during evolution [60,61,62]. Tan and colleagues showed that the transport protein particle (TRAPP) III complex may be recruited to the phagophore assembly site of COPII during macroautophagy and bound to SEC23, providing the membrane components to form autophagosomes [63]. Recently, a mechanism based on ULK-FBXW5-SEC23B action was described. It was demonstrated that F-Box and WD repeat domain containing 5 (FBXW5) may inhibit biogenesis of the COPII-mediated autophagosome by targeting and promoting SEC23B degradation in the presence of nutrients, whereas ULK1 phosphorylates SEC23B at S186 and prevents SEC23B and FBXW5 interaction in order to inhibit SEC23B degradation during cell starvation, culminating in cell autophagy [59].

The *TPR* gene was also modulated, and previous studies from our group have found that *BECN1*, *GPSM3*, *ATG14*, *APOL1*, and *TPR* genes are upregulated in PB cells compared to in MB cells. Interestingly, these five genes are also upregulated in T1R lesions. However, the *TPR* gene showed the greatest fold change when comparing samples of MB patients with and without T1R episodes [13]. *TPR* plays a role in autophagy through the control of Hsp70 and HSF1 mRNA export, p53 trafficking, and direct transcriptional regulation of autophagy factors [64,65]. Although preliminary, our data suggest that *TPR* could have a regulatory role in the induction of autophagy in both PB leprosy and during T1R episodes in MB patients. The *GPSM2* gene was also present in the list of 13 genes common between T1R skin lesions and cells stimulated by *M. leprae* + IFN-*γ*. *GPSM2* acts mainly in the regulation of the cell cycle and in the stability of cell division [66].

In summary, the data presented here indicate that autophagy modulation is associated with T1R pathogenesis in MB patients and that IFN-*γ*-stimuli in *M. leprae*-stimulated THP-1 cells may upregulate some genes that are expressed in the skin of T1R patients. Overall, these data suggest that identifying targets that modulate lysosomal/autophagic pathways during T1R may lead to the discovery of novel host-based therapeutic strategies to effectively control leprosy and T1R.

## Figures and Tables

**Table 1:** Baseline characteristics of leprosy patients included in the study.

	PB	MB No Progression	MB Progression	T1R
Male/female	6/8	7/1	5/2	9/3
Age, mean (range)	54.5 (8–92)	53.37 (34–65)	45.14 (32–69)	49.16 (17–66)
BI, mean (range)	0 (0–0)	2.15 (1.50–5.50)	2.98 (0.50–4.67)	2.66 (0.75–5.85)
LBI (range)	0 (0–0)	4.38 (2.85–5.95)	4.6 (2.7–5.95)	2.06 (0–3.8)
**Ridley–Jopling Clinical Form of Leprosy, *n***				
BT	14	-	-	-
BL	-	4	4	9
LL	-	4	3	3

## Data Availability

Data is contained within the article or Supplementary Material. Public microarray data is readily available in Gene Expression Omnibus (GSE74481). For details pertaining methods, please see Calvo and Moraes, 2020 (doi: 10.1007/s00438-020-01705-6).

## Supplementary Materials

The following are available online at https://www.mdpi.com/article/10.3390/cells10092215/s1, Figure S1: Immunoblot analysis of LC3 in THP-1 macrophages, Table S1: Autophagy gene expression signatures, Venn diagram overlaps and protein annotations, Figure S2: Transmission electron microscopy analysis of THP-1 macrophages, Figure S3: Autophagy gene-expression profiling of leprosy lesions.
