# Supplementary material for: Autophagy-Associated IL-15 Production Is Involved in the Pathogenesis of Leprosy Type 1 Reaction
Source: Cells. 2021 Aug 27;10(9):2215. doi: 10.3390/cells10092215 (PMC8468917; doi:10.3390/cells10092215)
Supplement: Supplementary file 1 [file cells-10-02215-s001.zip › cells-1225879 SI.pdf]

Supplementary Materials

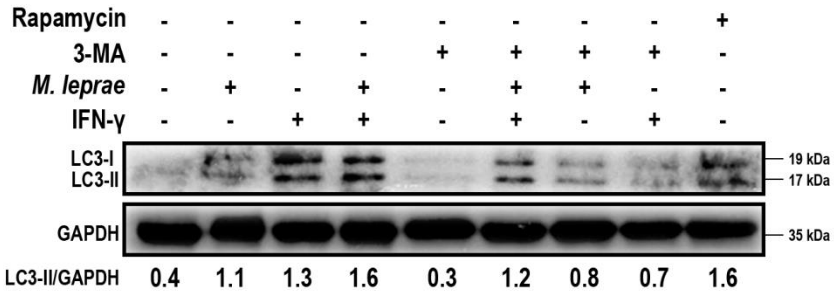

Figure S1: Immunoblot analysis of LC3 in THP-1 macrophages. THP-1 macrophages were differentiated for 24 hours in the presence of 200 nM PMA, pretreated with 10 mM 3-MA (3-methyladenine) for 1 hour, stimulated with *M. leprae* for 30 minutes (MOI 10:1) and treated with 10 ng/mL of IFNγ or 200 ng/mL of rapamycin for 18 hours. LC3 expression was assessed by Western blotting using an anti-LC3 antibody. GAPDH antibody was used to verify protein amount loading. Densitometric analysis was performed and the LC3-II/GAPDH ratio values are shown under the blots. Data are representative of 3 independent experiments performed.

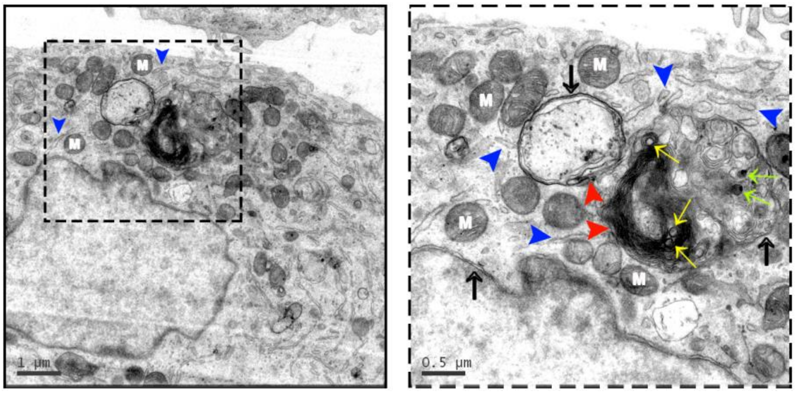

Figure S2: Transmission electron microscopy analysis of THP-1 macrophages. THP-1 macrophages were differentiated for 24 hours in the presence of 200 nM PMA, stimulated with M. leprae (MOI 10:1) for 30 minutes, then treated with 10 ng/mL of IFN-γ for 18 hours and processed for analysis by transmission electron microscopy. M, mitochondria; Blue arrowheads, endoplasmic reticulum; Black arrows, double-membrane (autophagosomes) visible sites; Red arrowheads, onion/myelin-like multilamellar structures; Green arrows, M. leprae; Yellow arrows, electron-lucent structures resembling damaged bacilli. The images are representative of 3 experiments.

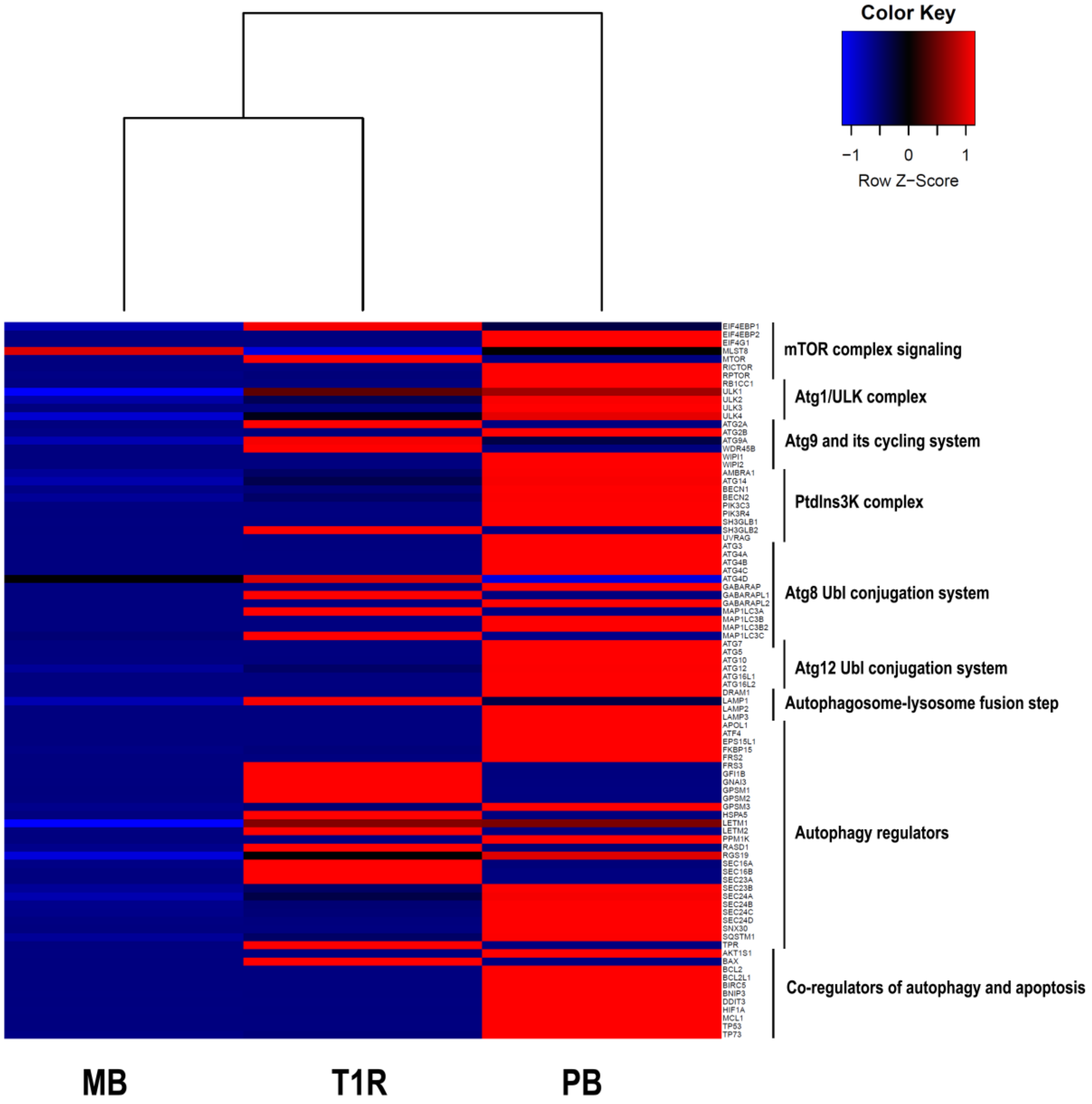

Figure S3: Autophagy gene-expression profiling of leprosy lesions. Purified mRNAs from skin lesions of type 1 reaction (T1R), multibacillary (MB) and paucibacillary patients (PB) were analyzed by RT-qPCR using an autophagy pathway array PCR kit. Heatmap shows the analysis of autophagy-related genes aggreged in different categories. Each line is representative of a gene. Data are representative of 4 (PB), 7 (MB) and 7 (T1R) samples.
